# Novel GSIP: GAN-based sperm-inspired pixel imputation for robust energy image reconstruction

**DOI:** 10.1038/s41598-024-82242-9

**Published:** 2025-01-07

**Authors:** Gamal M. Mahmoud, Wael Said, Magdy M. Fadel, Mostafa Elbaz

**Affiliations:** 1 Department of Electrical Engineering, Pharos University in Alexandria, Alexandria, Egypt; 2 Computer Science Department, Faculty of Computers and Informatics, Zagazig University, Zagazig, 44511 Egypt; 3 Computer Engineering and Systems Department, Faculty of Engineering, Mansoura University, Mansoura, 35516 DK Egypt; 4 Department of Computer Science, Faculty of Computers and Informatics, Kafrelsheikh University, Kafrelsheikh, Egypt

**Keywords:** Pixel imputation, GANs, Identity block, Intelligent sperm attitude, Energy source images, Solar fault detection, Image processing, Machine learning

## Abstract

Missing pixel imputation is a critical task in image processing, where the presence of high percentages of missing pixels can significantly degrade the performance of downstream tasks such as image segmentation and object detection. This paper introduces a novel approach for missing pixel imputation based on Generative Adversarial Networks (GANs). We propose a new GAN architecture incorporating an identity module and a sperm motility-inspired heuristic during filtration to optimize the selection of pixels used in reconstructing missing data. The intelligent sperm motility heuristic navigates the image’s pixel space, identifying the most influential neighboring pixels for accurate imputation. Our approach includes three essential modifications: (1) integration of an identity module within the GAN architecture to mitigate the vanishing gradient problem; (2) introduction of a metaheuristic algorithm based on sperm motility to select the top 10 pixels that most effectively contribute to the generation of the missing pixel; and (3) the implementation of an adaptive interval mechanism between the discriminator’s actual value and the weighted average of the selected pixels, enhancing the generator’s efficiency and ensuring the coherence of the imputed pixels with the surrounding image context. We evaluate the proposed method on three distinct datasets (Energy Images, NREL Solar Images, and NREL Wind Turbine Dataset), demonstrating its superior performance in maintaining pixel integrity during the imputation process. Our experiments also confirm the approach’s effectiveness in addressing everyday challenges in GANs, such as mode collapse and vanishing gradients, across various GAN architectures.

## Introduction

In image processing, the integrity of pixel data is paramount for the success of various tasks, including image segmentation, object detection, and image reconstruction^1–5^. Missing pixels, whether due to noise, sensor errors, or data corruption, can severely impair the performance of these tasks. Imputation of missing pixels is a critical preprocessing step aimed at restoring the lost information to ensure accurate and reliable downstream analysis^6,7^.

Traditional methods for missing pixel imputations, such as interpolation and inpainting, often fall short in complex scenarios where large portions of pixel data are missing or where the surrounding pixel context is insufficient for accurate reconstruction^8,9^. These methods struggle with maintaining images’ fine details and structural coherence, leading to artifacts that negatively impact subsequent image processing tasks^10^.

Generative Adversarial Networks (GANs) have emerged as powerful tools in image processing, particularly in tasks involving image synthesis, enhancement, and imputation^11^. GANs are designed to generate new data points indistinguishable from the original data, making them highly suitable for reconstructing missing pixels. However, standard GAN architectures have limitations, especially when dealing with high percentages of missing data^12,13^.

One of the critical challenges GANs face, particularly during the training process, is the vanishing gradient problem^14^. This issue occurs when the gradient updates become exceedingly small, hindering the generator network’s learning process^15^. This problem is exacerbated in deep architectures, where the information from the loss function fails to propagate effectively back through the network layers. Addressing this problem is essential for improving GANs’ performance in tasks like missing pixel imputation.

Another significant challenge in GANs is mode collapse^16^, where the generator fails to capture the full diversity of the data distribution, leading to the generation of similar or identical outputs^17^. This phenomenon is particularly detrimental in missing pixel imputations, where diverse and contextually accurate pixel generation is crucial. Addressing mode collapse is, therefore, a key focus in advancing GAN-based imputation methods.

This paper proposes a novel GAN architecture that integrates an identity module designed to tackle the vanishing gradient problem. The identity module ensures that the gradients are preserved across the network layers, facilitating more effective learning and improving the overall quality of the generated pixels. This architectural modification enhances the robustness of the GAN and contributes to solving the challenges associated with deep networks in pixel imputation tasks.

A unique aspect of our approach is incorporating a metaheuristic algorithm inspired by sperm motility, which guides the selection of pixels used in the imputation process. The sperm motility heuristic simulates the natural movement of sperm cells, known for their ability to navigate complex environments to reach their target. By mimicking this intelligent navigation, our algorithm identifies the most relevant and influential pixels that should be used to reconstruct missing data, thereby enhancing the accuracy and efficiency of the imputation process.

In addition to the identity module and sperm motility heuristic, we introduce a third innovation: an adaptive interval mechanism. This mechanism creates a dynamic interval between the actual value of the discriminator and the weighted average of the selected pixels. The adaptive interval plays a crucial role in improving the generator’s efficiency by reducing the time required for pixel generation while ensuring that the generated pixels are coherent with the surrounding image context. This mechanism speeds up the imputation process and enhances the overall quality of the imputed images.

To validate the effectiveness of our proposed approach, we conducted extensive experiments on three distinct datasets, each representing different types of image data and varying levels of complexity. Our results demonstrate significant improvements in pixel integrity and a marked reduction in common GAN-related issues such as mode collapse and vanishing gradients. The experimental outcomes provide strong evidence of the robustness and reliability of our method in addressing the challenges of missing pixel imputation.

Contributions of the Paper:

1. Introduces a novel sperm motility-inspired heuristic that emulates the natural movement patterns of sperm cells. This innovative approach facilitates intelligent navigation through pixel space, identifying the most influential neighboring pixels. The strategy significantly enhances the contextual coherence of imputed pixels, thereby improving overall image quality. Its versatility across diverse image processing applications, from medical imaging to computer vision, further underscores its potential impact and broad applicability.
2. The proposed methodology effectively addresses persistent challenges in Generative Adversarial Networks (GANs), such as mode collapse and the vanishing gradient problem, through specific architectural modifications, including integrating an identity block. This results in improved diversity and accuracy in image reconstruction compared to other GAN architectures, validated across three distinct datasets.
3. Enhances the accuracy and computational efficiency of missing pixel imputation, ensuring the high integrity of imputed pixels about the overall image structure, thereby facilitating reliable downstream analysis.
4. Comprehensive testing and case studies demonstrate the efficacy of each methodology component, including the sperm motility heuristic and the identity block, providing empirical evidence of their contributions to the proposed approach’s overall performance and efficiency.

The organization of this paper is structured as follows: Sect. 2 presents a detailed review of related work, examining existing approaches for missing pixel imputation and the application of Generative Adversarial Networks (GANs) in image processing, along with the challenges they face, such as the vanishing gradient problem and mode collapse. Section 3 outlines the proposed methodology, introducing our novel GAN architecture, integrating an identity module, the sperm motility-inspired metaheuristic, and the adaptive interval mechanism designed to enhance imputation accuracy and efficiency. Section 4 discusses the experimental results, comparing the performance of our approach with existing methods and analyzing its effectiveness in solving the identified challenges. Section 5 concludes the paper, summarizing the work’s key findings, contributions, and implications and offering insights into potential future research directions and applications of the proposed approach.

## Related work

Generative Adversarial Networks (GANs) have become a cornerstone in image processing, particularly in tasks requiring high-quality image synthesis, enhancement, and imputation. Since their introduction, GANs have been applied to various domains, including image inpainting, super-resolution, and, more recently, missing pixel imputation. This section reviews the most relevant advancements in GAN-based missing data imputation from 2021 to 2024, highlighting key contributions and identifying gaps our work aims to address.

In 2021, a study by Chen et al.^18^ explored a novel GAN architecture designed to improve missing data imputation in medical images. Their approach, MedGAN, incorporated domain-specific loss functions tailored to preserve anatomical correctness. Despite its success in the medical field, MedGAN struggled with generalizability when applied to non-medical datasets, particularly those with complex textures and patterns.

Qin et al.^19^ introduced MGAIN, a new missing data imputation method based on Generative Adversarial Networks (GAN). The process aims to overcome challenges such as gradient vanishing and mode collapse, common in existing GAN-based imputation methods. The new method improves the accuracy of missing data imputation by employing a least squares loss function to address gradient vanishing and a dual discriminator to prevent mode collapse. The authors stated that MGAIN outperformed cutting-edge methods, lowering the root mean square error by 21.66%.

The issue of missing data in multi-view datasets is addressed by Zhang et al.^20^ with a novel technique called VIGAN, which uses Generative Adversarial Networks (GANs). This technique is beneficial when a whole data view is missing for specific samples. Conventional methods such as matrix completion and multiple imputations are useless. To solve this problem, VIGAN treats each view as a distinct domain, uses GANs to map between domains, and then reconstructs the missing view using a multi-modal denoising autoencoder (DAE). Benchmark datasets and a genetic study validate the approach, demonstrating its usefulness in the life sciences and its ability to recover missing data.

Qu et al.^21^ developed a deep learning-based sound log data imputation method that combines Conditional Generative Adversarial Networks (CGAN) with swarm intelligence optimization algorithms to overcome the challenges of missing or incomplete sound log data in deep and ultra-deep oil and gas exploration. The method uses seismic layer velocity as a constraint to guide CGAN in producing realistic, well-log data corresponding to geological features. At the same time, the swarm intelligence algorithm optimizes the generation process. The approach outperforms other algorithms and provides a novel solution for sequence data prediction in oil and gas development.

Zhang et al.^22^ proposed a novel unified method for multi-modal medical image synthesis to address the issue of missing modalities in clinical imaging caused by limited scanning time, image corruption, or varying imaging protocols. The proposed method employs a generative adversarial architecture to generate missing modalities from any combination of existing ones using a single model. It introduces a Commonality- and Discrepancy-Sensitive Encoder that exploits modality-invariant and specific information, resulting in anatomically consistent and realistic images. Furthermore, a Dynamic Feature Unification Module is intended to robustly integrate a variety of available modalities while preventing information loss. The method is validated on public datasets and outperforms existing approaches.

Despite these advancements, existing GAN-based methods for missing pixel imputation often struggle with two persistent issues: the vanishing gradient problem and mode collapse^23–27^. The vanishing gradient problem, particularly prevalent in deep networks, hinders the practical training of GANs by diminishing gradient updates during backpropagation. Mode collapse, however, restricts the diversity of generated outputs, leading to repetitive and less varied imputation results.

Our proposed approach addresses these limitations through several innovative strategies. First, we introduce an identity block within the GAN architecture to mitigate the vanishing gradient problem, ensuring better gradient flow and more stable training. This approach is particularly novel in its application to missing pixel imputation, as it allows for more effective learning in deep networks. Our method also incorporates a metaheuristic algorithm inspired by sperm motility, which intelligently selects the most influential neighboring pixels for imputation. This biologically inspired approach enhances the accuracy and context-awareness of the imputed pixels, setting it apart from traditional heuristic methods. We also introduce an adaptive interval mechanism that dynamically adjusts the interval between the discriminator’s actual value and the weighted average of selected pixels. This mechanism accelerates the imputation process and ensures that the imputed pixels are coherent with the surrounding image context, reducing artifacts and improving overall image quality. While our method significantly improves the imputation process, it has challenges. The integration of a sperm motility-inspired metaheuristic, although innovative, introduces additional computational complexity, which may limit its scalability for large-scale datasets. Moreover, while effective in maintaining pixel coherence, the adaptive interval mechanism requires careful calibration to avoid overfitting, particularly in datasets with high variability.

## The methodology

The core of the proposed approach is a Generative Adversarial Network (GAN) architecture, which serves as the framework for the missing pixel imputation task. The GAN model consists of two key components: the Generator and the Discriminator. The Generator network is responsible for producing the imputed pixel values to fill in the missing regions of the input image. A unique aspect of the Generator network, and a crucial innovation introduced in this work, is the inclusion of an ‘Identity’ block. This novel architectural component addresses the vanishing gradient problem, a common issue encountered in deep neural networks. By integrating the Identity block within the Generator, the paper aims to improve the gradient flow throughout the network, ensuring more stable and effective training of the GAN model. In addition to the Identity block, the Generator network also utilizes a metaheuristic approach inspired by sperm motility to guide the selection of the most influential neighboring pixels during the imputation process. This biologically inspired technique mimics the natural movement patterns of sperm cells, which are known for their ability to navigate complex environments to reach their target. By leveraging this intelligent navigation, the algorithm identifies the most relevant and impactful pixels to be used in the reconstruction of missing data, enhancing the accuracy and contextual coherence of the imputed pixels.

**Fig. 1:**
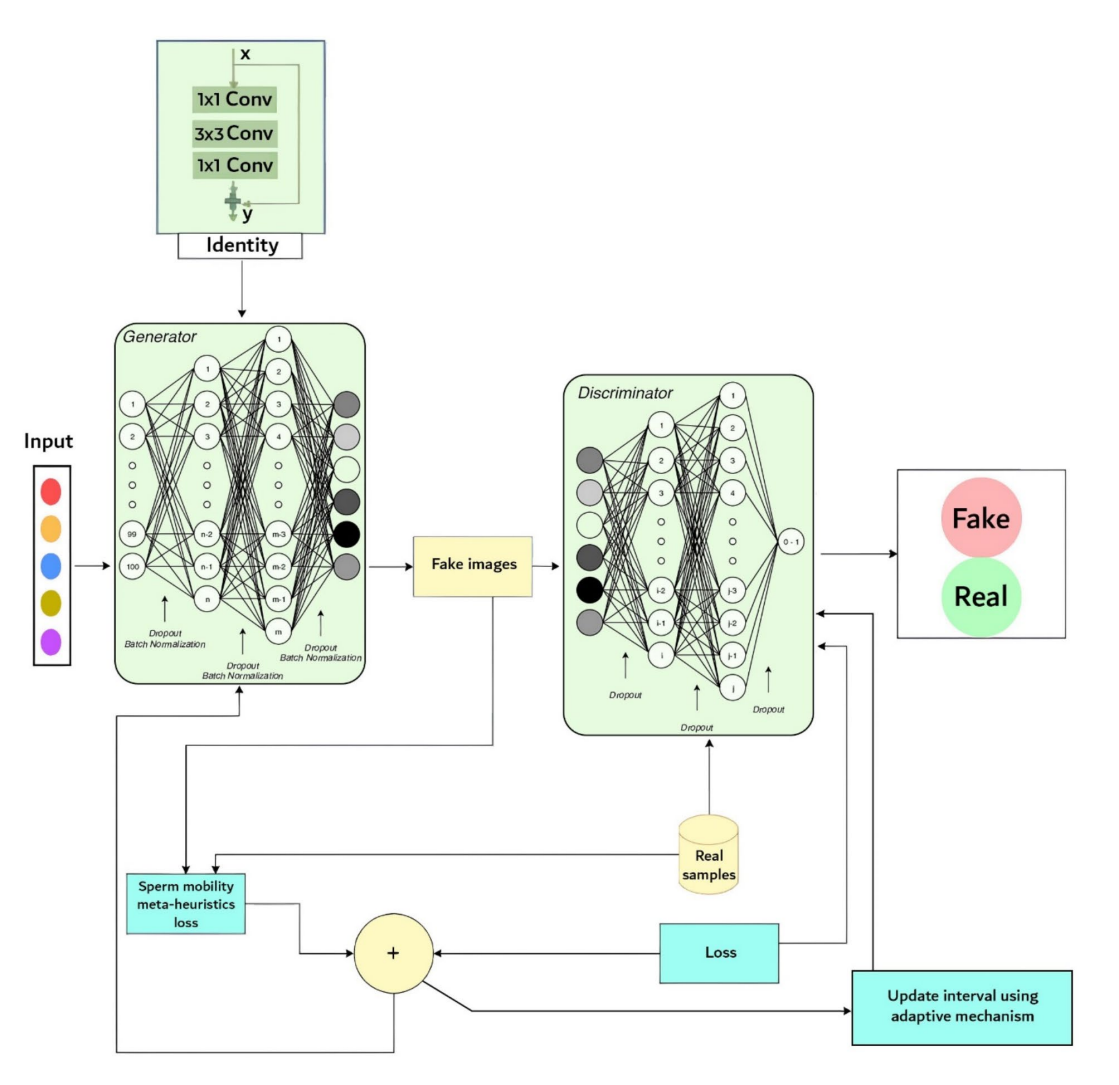
Block diagram of the methodology.

Figure 1 shows the block diagram of the methodology. The block diagram begins with an “X” input block, representing the image with missing pixels that need to be imputed. This input is then passed through an “Identity” block, a novel architectural component introduced in the paper. Including the Identity block within the Generator network of the GAN is a crucial innovation designed to address the vanishing gradient problem, a common issue in deep neural networks.

The Generator network is responsible for producing the imputed pixel values to fill in the missing regions of the input image. A unique aspect of the Generator is the incorporation of a metaheuristic approach inspired by sperm motility. This biologically inspired technique guides the Generator in intelligently selecting the most influential neighboring pixels during the imputation process. By mimicking the natural movement patterns of sperm cells, the algorithm aims to enhance the accuracy and contextual coherence of the generated pixels, leading to more realistic and coherent imputation results. On the other side of the diagram, the Discriminator network evaluates the authenticity of the generated (fake) pixels by comparing them to real image samples. The Discriminator is also augmented with structural similarity metrics to ensure the visual quality and integrity of the imputed regions are maintained. This combination of adversarial training and structural similarity helps preserve the coherence and fidelity of the imputed pixels within the overall image context. The methodology also introduces an ‘Adaptive Interval Mechanism’ that dynamically adjusts the interval between the Discriminator’s real value and the weighted average of the selected pixels. This innovative mechanism plays a crucial role in improving the Generator’s efficiency, reducing the time required for pixel generation, and ensuring the coherence of the imputed pixels with the surrounding image context. By adaptively adjusting this interval, the approach prevents the introduction of visual artifacts and maintains the overall quality of the imputed image. The ‘Sperm motility meta-heuristics loss’ component serves as a guiding force for the pixel selection process within the Generator network. This biologically inspired approach, drawing from the natural movement patterns of sperm cells, helps the Generator optimize the selection of the most relevant neighboring pixels, leading to more accurate and contextually appropriate imputation results. The block diagram illustrates the novel architectural modifications and techniques proposed in the paper to address the challenges of missing pixel imputation using GANs, such as vanishing gradients, mode collapse, and maintaining image integrity. The algorithm 1 shows the main steps of the methodology.

**Algorithm 1:**
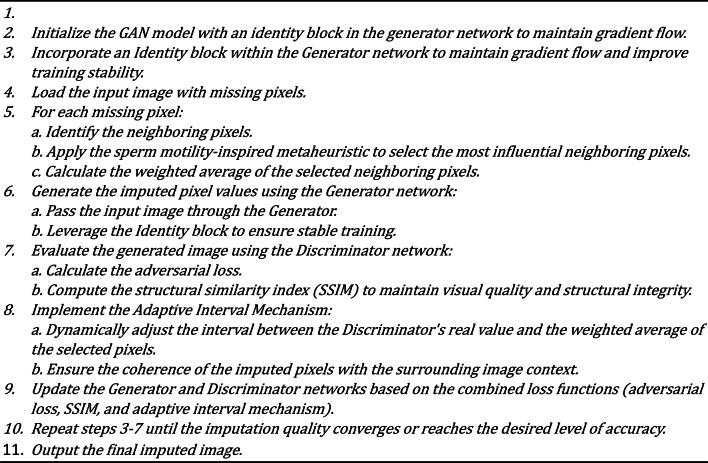
Main Steps of the Methodology

### The dataset

The paper evaluates the approach across three different datasets for energy images: Energy Images^28^, NREL Solar Images^29^, and NREL Wind Turbine^30^ Dataset. The Open Energy Images dataset is an extensive collection of over 240,000 annotated images covering various energy infrastructures and technologies, including power plants, renewable energy installations, and energy distribution networks. The NREL Solar Images dataset provides over 4,000 categorized and labeled images focused explicitly on solar photovoltaic systems. At the same time, the NREL Wind Turbine Dataset contains around 5,000 images of wind turbines with annotated bounding boxes. Together, these three datasets offer a comprehensive visual resource for researchers and practitioners working on energy-related computer vision and machine learning applications, enabling studying different energy generation, distribution, and utilization systems through high-quality, well-annotated image data. The breadth of coverage, detailed metadata, and accessibility of these datasets make them valuable tools for advancing research and development in the energy domain.

### GANs architecture with the identity block

The Generative Adversarial Network (GAN) is the core framework for missing pixel imputation in our methodology. The GAN architecture comprises two primary components: the Generator and the Discriminator. The Generator is responsible for producing plausible pixel values that can seamlessly fill in the missing regions of an image. At the same time, the Discriminator evaluates the authenticity of these generated pixels by distinguishing between the actual (original) and fake (imputed) pixels. Our GAN model is enhanced with an identity block within the Generator to ensure stable gradient flow, which addresses the vanishing gradient problem often encountered in deep networks. Additionally, the Discriminator is augmented with structural similarity metrics to preserve the visual quality and integrity of the images. This architecture enables the GAN to generate highly realistic and contextually appropriate imputations, even in challenging scenarios with irregularly missing data. Table 1 shows the architecture of GANs with the identity block. Algorithm 2 and Algorithm 3 show the architecture of the Generator and Discriminator, respectively.

The Identity block, a pivotal architectural component integrated within the Generator network of our GAN-based methodology for missing pixel imputation, plays a crucial role. It effectively addresses the common challenge of vanishing gradients, a problem that can arise during the training of deep neural networks. The core principle behind the Identity block is to create a direct shortcut connection that allows a portion of the input data to pass through the network without any transformation. This skip connection ensures that the gradients can flow more effectively through the network layers, preventing the gradients from vanishing as they propagate backward during the training process. By maintaining a stable gradient flow, the Identity block significantly enhances the overall training stability of the Generator network, enabling it to reliably converge to an optimal solution.

In the context of the missing pixel imputation task, the Identity block is seamlessly integrated within the Generator’s architecture. As the input image with missing pixels passes through the Generator network, the Identity block takes a portion of this input. It directly propagates it through the network, bypassing any transformations. This direct propagation of the input data, combined with the Generator’s learned transformations, helps preserve the essential contextual information required to generate accurate and coherent imputed pixel values. The synergistic interplay between the Identity block and the sperm motility-inspired metaheuristic is particularly noteworthy. The metaheuristic algorithm guides the Generator network in intelligently selecting the most influential neighboring pixels for the imputation process. By leveraging the natural navigation patterns of sperm cells, the metaheuristic can identify the pixel relationships and dependencies crucial for maintaining the structural integrity and coherence of the imputed regions. The Identity block, in turn, ensures that the gradients associated with these selected influential pixels can flow unimpeded through the Generator network. This allows the Generator to effectively learn the necessary transformations to generate imputed pixels that seamlessly integrate with the surrounding image context, preserving the overall visual quality and authenticity. Combining the Identity block’s stable gradient propagation and the sperm motility-inspired metaheuristic’s intelligent pixel selection creates a powerful and synergistic approach to missing pixel imputation. This holistic methodology helps overcome common challenges in GAN-based image generation, resulting in superior performance in terms of the imputed pixels’ accuracy and visual coherence.

**Table 1:** Architecture of the GANs architecture with identity block.

Layer	Generator	Discriminator
Input	Image with missing pixels	Full image (real or generated)
Convolutional Layer 1	64 filters, 3 × 3 kernel, ReLU, stride 1	64 filters, 3 × 3 kernel, Leaky ReLU, stride 2
Convolutional Layer 2	128 filters, 3 × 3 kernel, ReLU, stride 2	128 filters, 3 × 3 kernel, Leaky ReLU, stride 2
Batch Normalization 1	Applied after Conv Layer 2	Applied after Conv Layer 2
Convolutional Layer 3	256 filters, 3 × 3 kernel, ReLU, stride 2	256 filters, 3 × 3 kernel, Leaky ReLU, stride 2
Identity Block	Skip connection with 2 × 3 × 3 Conv, ReLU	Not Applicable
Batch Normalization 2	Applied after Identity Block	Applied after Conv Layer 3
Deconvolutional Layer 1	128 filters, 3 × 3 kernel, ReLU, stride 2	Not Applicable
Deconvolutional Layer 2	64 filters, 3 × 3 kernel, ReLU, stride 2	Not Applicable
Output Layer	3 channels, 3 × 3 kernel, Sigmoid	1 unit (real/fake), Sigmoid

**Algorithm 2:**
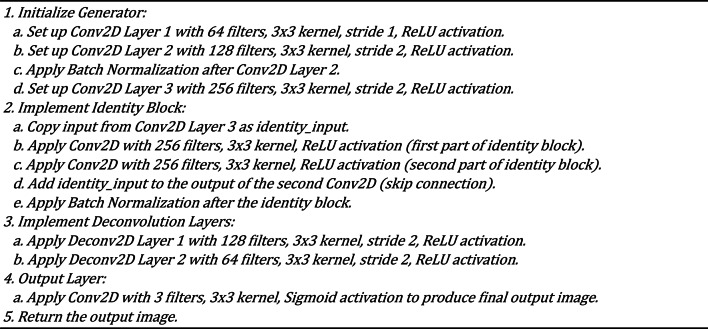
Generator pseudocode

**Algorithm 3:**
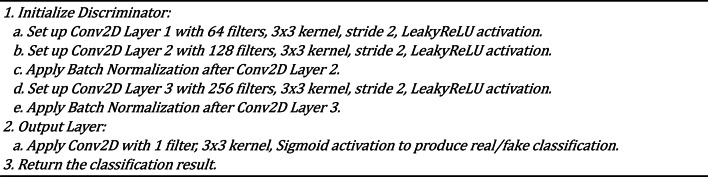
Discriminator pseudocode

### Motility attitude during filtration approach

The sperm motility-inspired metaheuristic is a unique and innovative component of the proposed approach for missing pixel imputation. This metaheuristic algorithm draws inspiration from the natural movement patterns of sperm cells, which are known for their remarkable ability to navigate complex environments to reach their target destination. In the context of the missing pixel imputation task, the sperm motility-inspired metaheuristic is used to intelligently select the most influential neighboring pixels that should be used to reconstruct the missing pixel values. This is a crucial step, as the quality and coherence of the imputed pixels directly depend on selecting the appropriate neighboring pixels. The core idea behind the sperm motility-inspired metaheuristic is to mimic the way sperm cells navigate through their environment. Sperm cells are guided by various chemical and physical cues, which allow them to efficiently navigate towards the sperm. Similarly, the proposed metaheuristic algorithm leverages the inherent patterns and relationships within the image data to identify the most influential neighboring pixels for the imputation process.

The process begins with the Identification of Neighboring Pixels. For each missing pixel, the algorithm first identifies the neighboring pixels that could potentially contribute to imputing the missing value. This involves examining the pixel’s spatial vicinity and considering the pixels close. The Evaluation of Pixel Influence takes place. The metaheuristic evaluates the influence of each neighboring pixel based on a set of criteria that mimic the navigation strategies of sperm cells. These criteria may include factors such as the spatial proximity of the adjacent pixel, its intensity value, and its structural similarity to the surrounding pixels. The algorithm assigns a weight to each neighboring pixel based on how well it aligns with these sperm-inspired evaluation criteria.

**Fig. 2:**
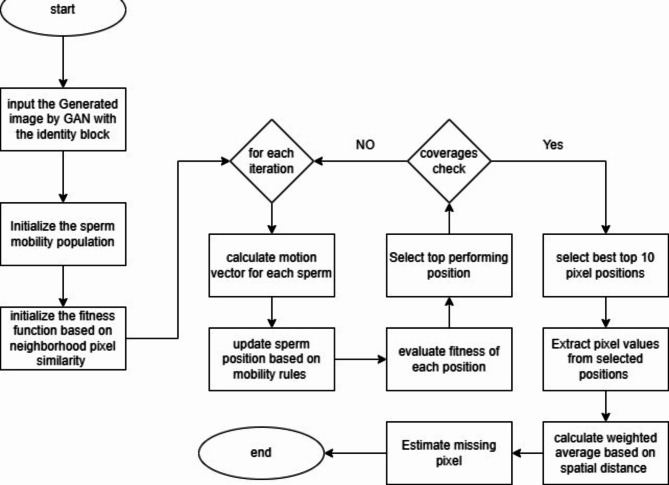
sperm mobility metaheuristic algorithm flow chart.

Building upon the pixel influence evaluation, the algorithm proceeds to select Top Influential Pixels. The algorithm determines the top 10 (or a configurable number of) most influential neighboring pixels based on the evaluation criteria. These selected pixels are the ones that are deemed to be the most relevant and impactful for the accurate imputation of the missing pixel. Finally, the Weighted Averaging step is performed. A weighted average of the selected neighboring pixels generates the imputed pixel value. The weighting scheme is designed to give more emphasis to the pixels that are deemed more influential by the metaheuristic algorithm. This weighted averaging helps to ensure that the generated pixel value is coherent with the surrounding image context, preventing the introduction of visual artifacts and maintaining the overall structural integrity of the image. The integration of this sperm motility-inspired metaheuristic within the overall methodology is crucial. By leveraging the intelligent pixel selection approach, the Generator network in the GAN framework can produce high-quality imputed pixels that effectively capture the underlying patterns and relationships within the image data. This, in turn, enables the Discriminator network better to evaluate the authenticity and coherence of the generated pixels, leading to a more robust and reliable image imputation process. The biologically inspired nature of the sperm motility-inspired metaheuristic also helps to overcome common challenges in GANs, such as mode collapse and vanishing gradients. By mimicking the natural navigation strategies of sperm cells, the algorithm can guide the Generator network towards more effective and stable pixel generation, ultimately resulting in superior performance in missing pixel imputation tasks. Algorithm 4 shows how the motility sperm meta-heuristics algorithm chooses the pixels that can be used to impute the missed pixels. Algorithm 5 shows the method of calculating the weighted average based on the sperm mobility meta-heuristics algorithm. Figure 2 show the flowchart sperm mobility metaheuristic algorithm.

**Algorithm 4:**
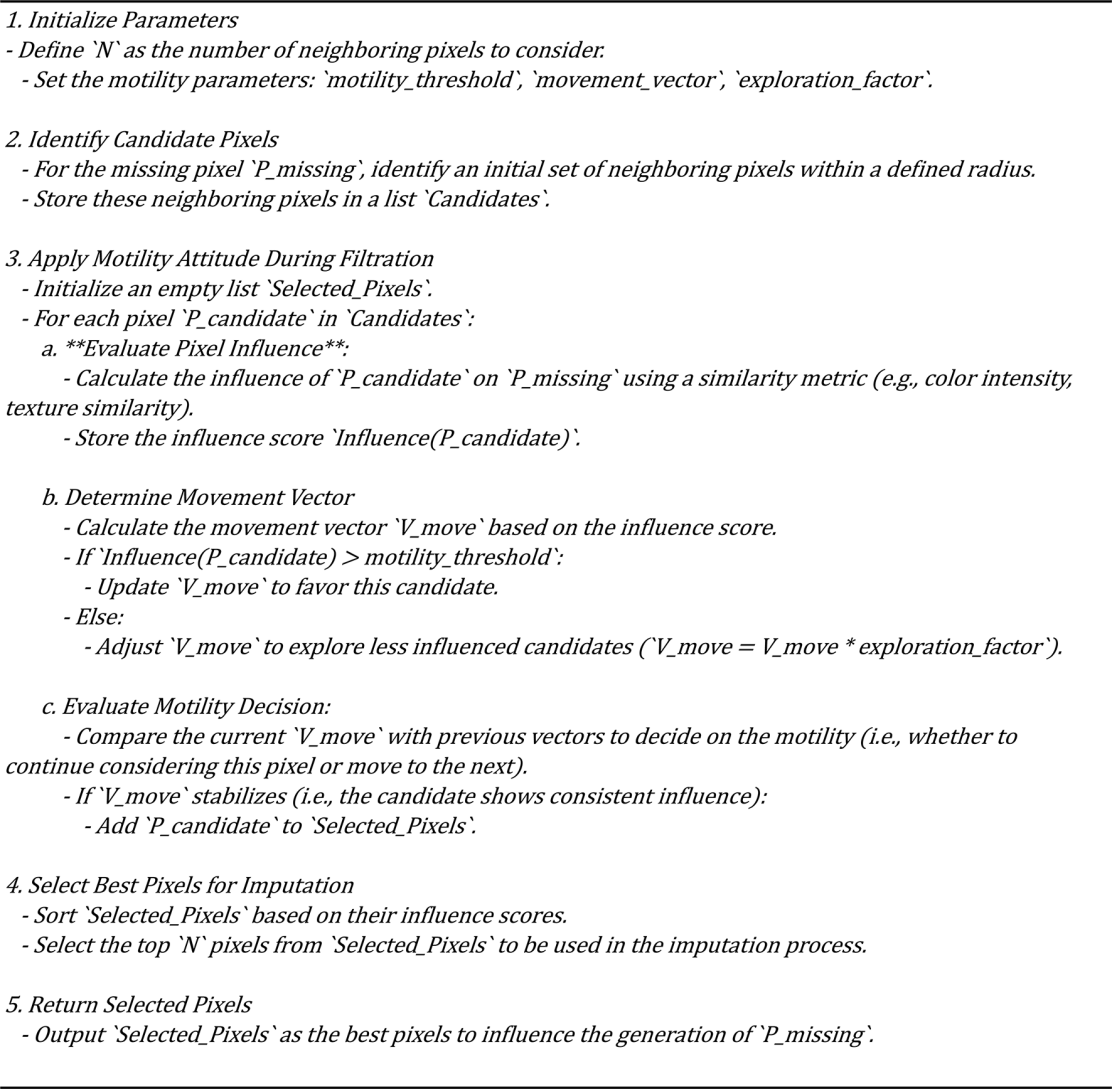
Motility Attitude During Filtration approach

**Algorithm 5:**
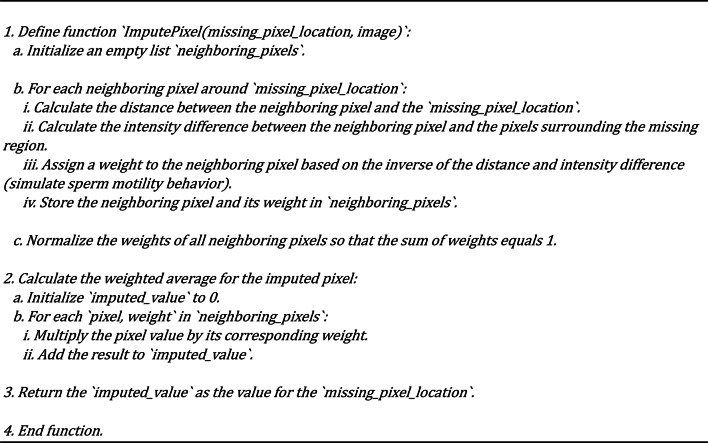
Weighted average pixel calculation

The sperm motility-inspired metaheuristic presented in the block diagram offers several advantages over other biological metaheuristic algorithms in the context of missing pixel imputation. One key advantage is the intelligent navigation capabilities of the approach, which draws inspiration from the natural movement patterns of sperm cells. Sperm cells are known for their remarkable ability to navigate complex environments and reach their target destination. The proposed metaheuristic can identify and select the most influential neighboring pixels for the imputation process by mimicking this intelligent navigation. This strategic selection of pixels helps enhance the imputed values’ accuracy and contextual coherence, leading to more realistic and visually appealing image reconstruction. Another notable advantage of the sperm motility-inspired metaheuristic is its adaptability to the specific domain of image-processing tasks. Many biological metaheuristics are designed for general optimization problems. Still, they may not be well-suited for the unique challenges of missing pixel imputation, where selecting relevant neighboring pixels is crucial for maintaining the integrity and structure of the image. The domain-specific adaptation of the proposed approach allows it to leverage the inherent patterns and relationships within the image data, enabling more effective performance in the context of image reconstruction. Integrating the sperm motility-inspired metaheuristic within the GAN framework also creates a synergistic effect, leveraging the strengths of both the biologically inspired pixel selection and the adversarial training process. The metaheuristic guides the Generator network to select the most influential pixels, while the Discriminator network evaluates the coherence and authenticity of the generated pixels. This combination helps to overcome common issues in GANs, such as mode collapse and vanishing gradients, leading to more robust and reliable image imputation.

### Adaptive interval mechanism of GANs with the identity block

Implementing an adaptive interval mechanism between the discriminator’s actual value and the weighted average of the selected pixels in our methodology enhances the robustness and accuracy of the imputation process. This mechanism dynamically adjusts the interval based on the evolving characteristics of the input data and the imputation context. By incorporating this adaptive strategy, the model ensures that the generated pixel is coherent with the immediate surrounding pixels and aligns well with the broader image context. The discriminator’s real value is a reference point, guiding the generator to produce more realistic and contextually appropriate pixels. Meanwhile, the weighted average of the selected pixels, informed by the Motility Attitude During the Filtration approach, offers a nuanced understanding of the local pixel environment. The adaptive interval bridges these two aspects, allowing the model to fine-tune the imputation process in real time, reducing the risk of generating artifacts or inconsistencies. This leads to a more seamless integration of the imputed pixels, enhancing the overall quality and realism of the reconstructed image.

Algorithm 6 shows the implementation of an adaptive interval mechanism that optimizes the imputation of missing pixels by dynamically adjusting the relationship between the discriminator’s actual value and the weighted average of selected pixels. The process begins with initializing critical parameters, including the interval that will be adjusted based on the model’s performance. The input image with missing pixels is then processed, where the best neighboring pixels are identified using the Motility Attitude During Filtration approach. These selected pixels compute a weighted average, the initial estimate for the imputed pixel.

The imputed pixel is generated using the generator network and is then evaluated by the discriminator to obtain a real value. The core of the mechanism lies in calculating the difference between this real value and the weighted average. If this difference exceeds the adaptive interval, the weighted average is adjusted towards the discriminator’s real value, ensuring that the generated pixel aligns more closely with the surrounding context. The adaptive interval, a key component, is fine-tuned throughout the process, expanding or contracting based on the model’s performance (loss function), which is continuously monitored. This iterative process continues until the imputation stabilizes, ensuring that the final output is both realistic and contextually accurate, thereby demonstrating the model’s adaptability and reliability.

The pseudocode’s architecture is based on a GAN framework enhanced with an adaptive interval mechanism and a biologically inspired pixel selection process. The generator and discriminator form the core components of the GAN. The generator produces imputed pixel values based on input from selected neighboring pixels. In contrast, the discriminator evaluates these generated pixels against real ones to guide the generator’s learning process.

A key innovation in this architecture is the integration of the Motility Attitude During the Filtration approach, which intelligently selects neighboring pixels to influence the imputation. This selection process is critical as it ensures that the pixels chosen for imputing the missing ones are contextually relevant and contribute meaningfully to the overall image quality.

The adaptive interval mechanism acts as a bridge between the discriminator’s evaluations and the generator’s outputs. By dynamically adjusting the interval based on the model’s loss, the architecture ensures that the imputed pixels are not only visually coherent but also statistically aligned with the real data distribution. This approach prevents overfitting or underfitting by maintaining a balance between exploration and exploitation during the training process, instilling confidence in the model’s learning process. As a result, the architecture is capable of producing high-quality, realistic imputations that are well-integrated with their surroundings, leading to superior performance in reconstructing images with missing data.

**Algorithm 6:**
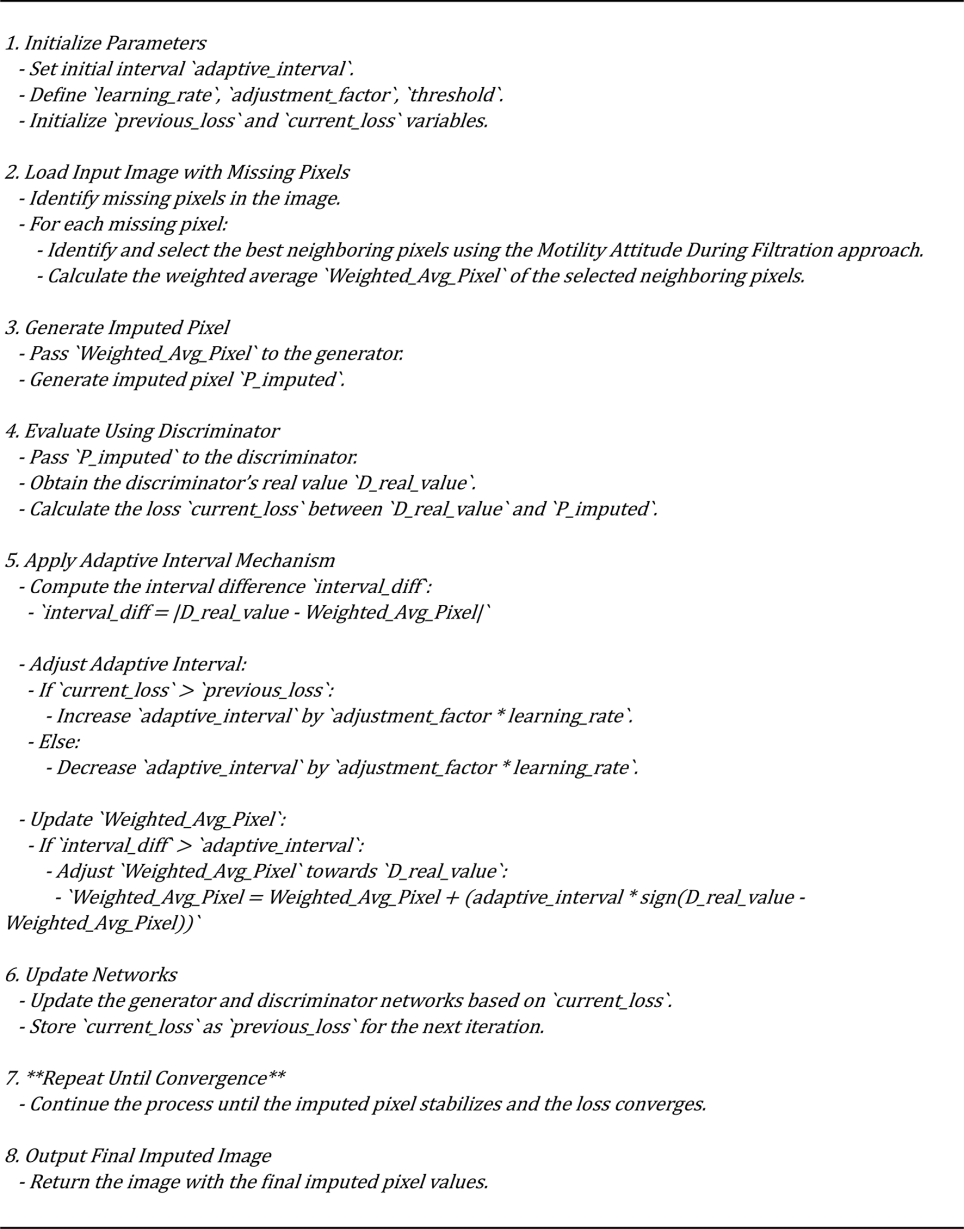
Weighted average pixel calculation.

### The evaluation metrics

In our methodology, we employ several well-established metrics to comprehensively evaluate the performance of our proposed generative model for missing pixel imputation. First, we calculate the Root Mean Squared Error (RMSE) between the generated pixel values and the ground-truth, as shown in Eq. (1). The RMSE provides a direct measurement of the reconstruction accuracy, allowing us to quantify how closely the generated pixels match the actual pixel values in the original images. Additionally, we compute the Inception Score (IS), defined in Eq. (2), to assess the quality and diversity of the reconstructed images. The IS metric leverages a pre-trained Inception model to capture the semantic information and class-conditional probability distributions of the generated pixels, giving us insights into the fidelity and visual coherence of the imputed regions. Finally, we measure the Fréchet Inception Distance (FID), as described in Eq. (3), to evaluate the similarity between the feature representations of the generated and real, complete images. The FID considers both the mean and covariance of the feature distributions, providing a holistic assessment of how closely the reconstructed images match the characteristics of the original data. Together, these complementary metrics allow us to thoroughly evaluate the efficacy of our proposed generative model for the critical task of missing pixel imputation.

1 $$\:RMSE=\sqrt[2]{{\sum\:}_{i=o}^{n}\frac{\:\left(genrated\:value-actual\:value\right)^2}{n}}$$

2 $$\:\text{I}\text{S}=\text{e}\text{x}\text{p}(\text{E}\text{x}\sim\:\text{p}\text{g}\text{D}\text{K}\text{L}(\text{p}\left(\text{y}\right|\text{x}\left)\right|\left|\text{p}\right(\text{y}\left)\right)).\:\:\:\:\:\:\:\:\:\:\:\:\:\:\:\:\:\:\:\:\:\:\:\:\:\:\:\:\:\:\:\:\:\:\:\:\:\:\:\:\:\:\:\:\:\:\:\:\:\:\:\:\:\:\:$$

3 $$\:\text{F}\text{I}\text{D}=\parallel\:{\upmu\:}\text{r}-{\upmu\:}\text{g}\parallel\:2+\text{T}\text{r}({\Sigma\:}\text{r}+{\Sigma\:}\text{g}-2({\Sigma\:}\text{r}{\Sigma\:}\text{g})1/2)).\:\:\:\:\:$$

## The results and discussion

This section of the paper presents the results of both imputation and diversity of GSIP-GAN for the (Open Energy Images, NREL Solar Images, and NREL Wind Turbine Dataset) datasets. The results also compare GSIP-GAN and different GAN architectures for imputation and diversity such as Wasserstein GAN (WGAN)^31^, Spectral Normalized GAN (SNGAN)^32^, Progressive Growing of GANs (PGGAN)^33^, Cycle-Consistent GANs (CycleGAN)^34^, and Spatially Adaptive Normalization (SPADE)^35^. An ablation study was conducted to highlight the effectiveness of various modifications in pixel imputation and image diversity. This study specifically tested the traditional GANs with enhancements, including the identity block and the sperm motility algorithm, individually and in combination with other integrated modifications. The results from this ablation study demonstrate that each enhancement contributes significantly to the model’s overall performance, reinforcing the importance of these targeted modifications in improving both the accuracy and diversity of the generated images. The results and discussion section also introduces a case study for detecting the fault in the solar panel using different new models before and after using our model.

### Results of imputation process

This section of the paper presents the comparison between GSIP-GAN and Wasserstein GAN (WGAN), Spectral Normalized GAN (SNGAN), Progressive Growing of GANs (PGGAN), Cycle-Consistent GANs (CycleGAN), and Spatially Adaptive Normalization (SPADE) using three different metrices for the imputation process. Table 2 showcases the Root Mean Squared Error (RMSE) comparison, which is a metric used to evaluate the accuracy of image reconstruction. The results indicate that the proposed “Our” model outperforms the other GAN models, consistently achieving the lowest RMSE values across all three datasets. This suggests that the “Our” model is highly effective in accurately reconstructing the missing or corrupted pixels in the energy-related images.

**Fig. 3:**
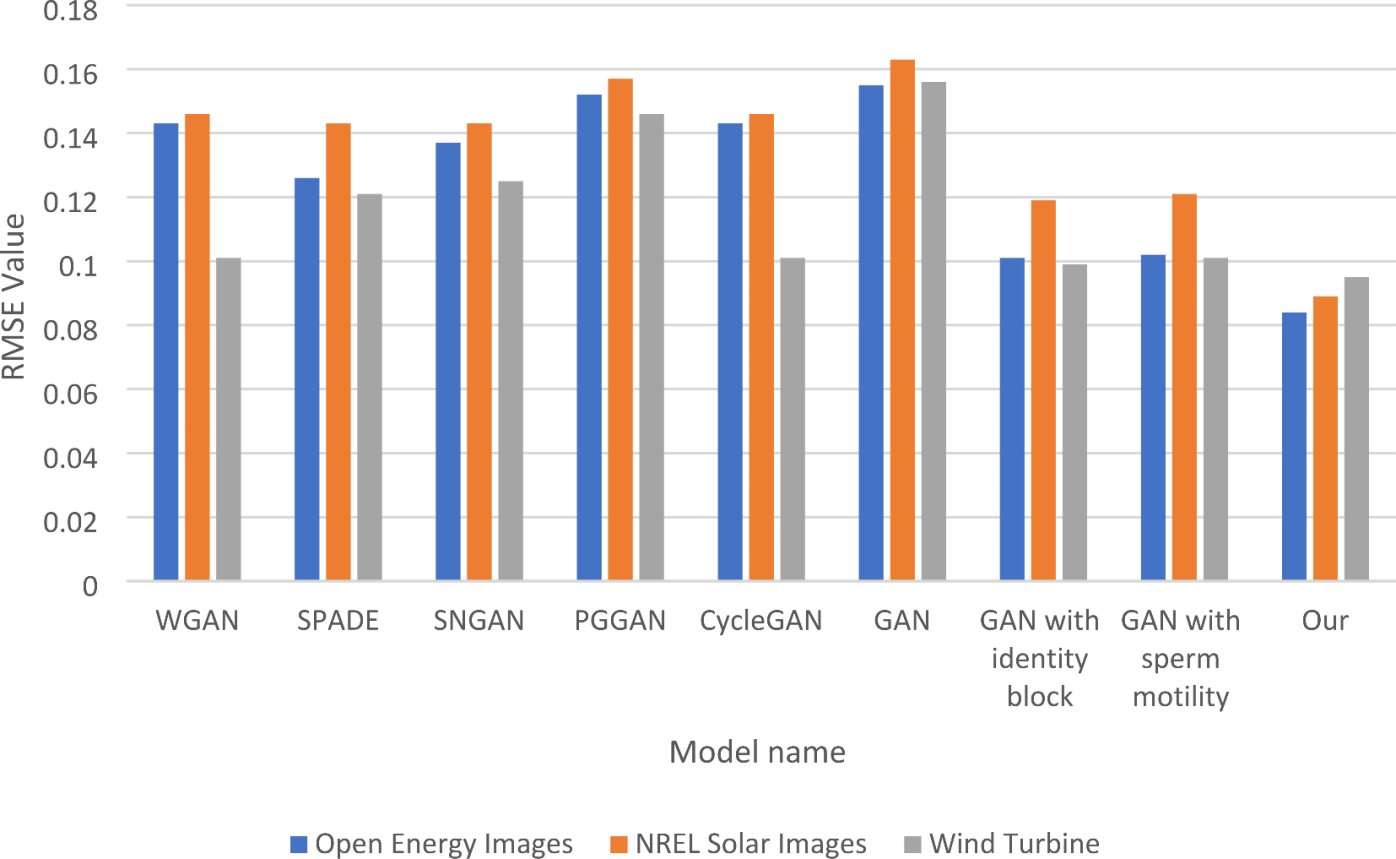
RMSE Comparison of five Models and GSIP-GAN Across three Datasets.

In Table 2, the RMSE comparison highlights the effectiveness of the ablation tests conducted on various GAN models across three datasets: Open Energy Images, NREL Solar Images, and Wind Turbine. The results indicate that “Our model,” which integrates all enhancements, achieves the lowest RMSE values—0.084 for Open Energy Images, 0.089 for NREL Solar Images, and 0.095 for Wind Turbine—demonstrating its superior performance. In contrast, the traditional GAN model exhibits relatively high RMSE values, particularly with an RMSE of 0.163 for NREL Solar Images, suggesting significant challenges in this dataset. The ablation tests reveal the contributions of specific modifications; for instance, the GAN with the identity block shows an RMSE of 0.101 on Open Energy Images, while the GAN with the sperm motility algorithm achieves 0.102. These results illustrate that while both modifications improve performance compared to the traditional GAN, neither independently surpasses the comprehensive enhancements of “Our model.” Overall, the findings underscore the importance of these targeted modifications, as they lead to significant reductions in RMSE, and highlight the need for further exploration of how these enhancements can be optimized for even better performance across diverse datasets. Figure 3 shows the Comparison of five Models and GSIP-GAN Across three Datasets in the term of RMSE. The last four models show the results of the traditional GAN, GAN after adding the identity block, GAN after adding the sperm mobility meta-heuristic algorithm and our integrated model, the results show the efficiency of each modification alone and the efficiency of all integrated modifications.

**Table 2:** RMSE comparison of five models and GSIP-GAN across three datasets.

Model	Open energy images	NREL solar images	Wind Turbine
Dataset			
WGAN	0.143	0.146	0.101
SPADE	0.126	0.143	0.121
SNGAN	0.137	0.143	0.125
PGGAN	0.152	0.157	0.146
CycleGAN	0.143	0.146	0.101
GAN	0.155	0.163	0.156
GAN with identity block	0.101	0.119	0.099
GAN with sperm motility	0.102	0.121	0.101
Our	0.084	0.089	0.095

In Table 3, the RSNR (Relative Signal-to-Noise Ratio) comparison reveals the performance of various GAN models across three datasets: Open Energy Images, NREL Solar Images, and Wind Turbine. The results indicate that “Our model” achieves the highest RSNR values, with 66.54 for Open Energy Images, 65.91 for NREL Solar Images, and 64.67 for Wind Turbine, highlighting its superior capability in preserving signal integrity. In contrast, the traditional GAN model shows significantly lower RSNR values, particularly with an RSNR of 50.43 for Open Energy Images, indicating a substantial loss of signal quality.

**Fig. 4:**
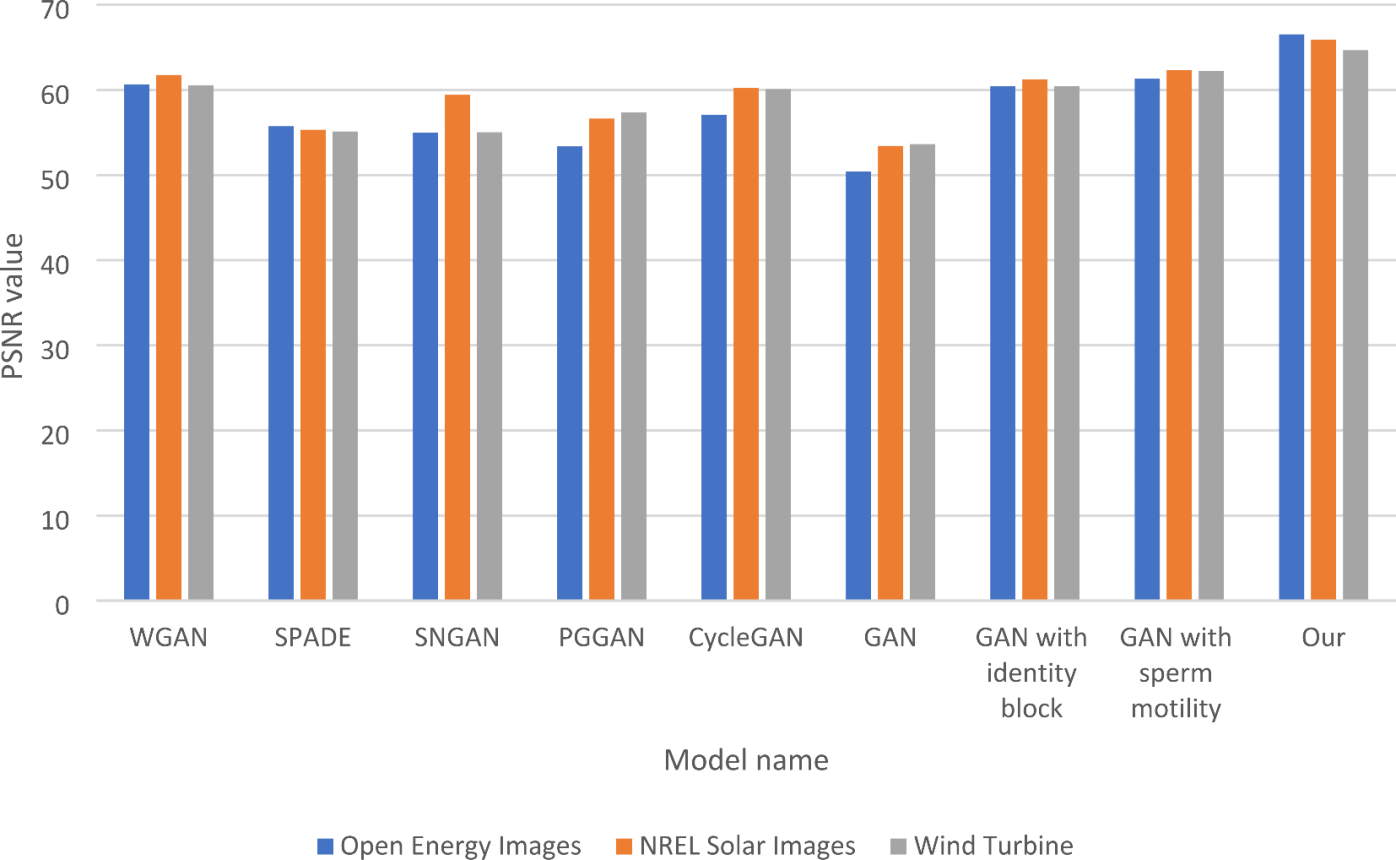
RSNR Comparison of five Models and GSIP-GAN Across three Datasets.

The ablation tests for the GAN models provide valuable insights into the contributions of specific enhancements. The traditional GAN serves as the baseline, exhibiting the lowest performance across both RMSE and RSNR metrics, which indicates challenges in effectively generating high-quality outputs. In contrast, incorporating the identity block leads to noticeable improvements, with the GAN achieving RSNR values around 60.43 across the datasets, suggesting enhanced feature preservation during training. Similarly, the GAN with sperm motility, which employs a meta-heuristic approach, achieves RSNR values of 61.32 for Open Energy Images and 62.32 for NREL Solar Images, reflecting practical signal preservation and better overall quality than the traditional GAN. “Our model,” which integrates the identity block and the sperm motility algorithm alongside other enhancements, clearly outperforms the different configurations, showcasing the highest RSNR values. This underscores the significance of combining these modifications, as they work synergistically to enhance signal integrity and accuracy. Overall, the findings in Table 3 highlight the effectiveness of the enhancements applied in “Our model” and suggest further exploration of these techniques to optimize performance across diverse datasets. Figure 4 shows the RSNR Comparison of five Models and GSIP-GAN Across three Datasets. Figure 4 also shows each step of improvement after adding the identity block, sperm mobility meta-heuristics algorithm, and the integrated models; the last four groups of columns show the enhancement for the traditional GANs after adding the identity block and sperm mobility.

**Table 3:** RSNR comparison of five models and GSIP-GAN across three datasets.

Model	Open Energy Images	NREL Solar Images	Wind Turbine
Dataset			
WGAN	60.65	61.76	60.52
SPADE	55.74	55.32	55.12
SNGAN	54.98	59.43	55.03
PGGAN	53.37	56.63	57.35
CycleGAN	57.09	60.23	60.1
GAN	50.43	53.43	53.64
GAN with identity block	60.43	61.23	60.43
GAN with sperm motility	61.32	62.32	62.23
Our	66.54	65.91	64.67

In Table 4, the SSIM (Structural Similarity Index Measure) comparison assesses the performance of various GAN models across three datasets: Open Energy Images, NREL Solar Images, and Wind Turbine. The results show that “Our model” achieves the highest SSIM scores, with 79.34 for Open Energy Images, 75.33 for NREL Solar Images, and 76.32 for Wind Turbine. This indicates a superior ability to preserve the generated outputs’ structural information and visual quality. The traditional GAN model, in contrast, exhibits lower SSIM scores, particularly with a value of 61.32 for Open Energy Images, highlighting its limitations in maintaining structural fidelity. The SSIM scores for the other models, such as WGAN and CycleGAN, are also relatively low, with WGAN achieving 72.32 for Open Energy Images and 70.11 for NREL Solar Images. These results demonstrate that while these models perform better than the traditional GAN, they still fall short of the performance achieved by “Our model.” The ablation tests further elucidate the contributions of specific enhancements. The GAN with the identity block matches the WGAN’s performance on Open Energy Images, scoring 72.32, and also shows improved results for the other datasets.

**Fig. 5:**
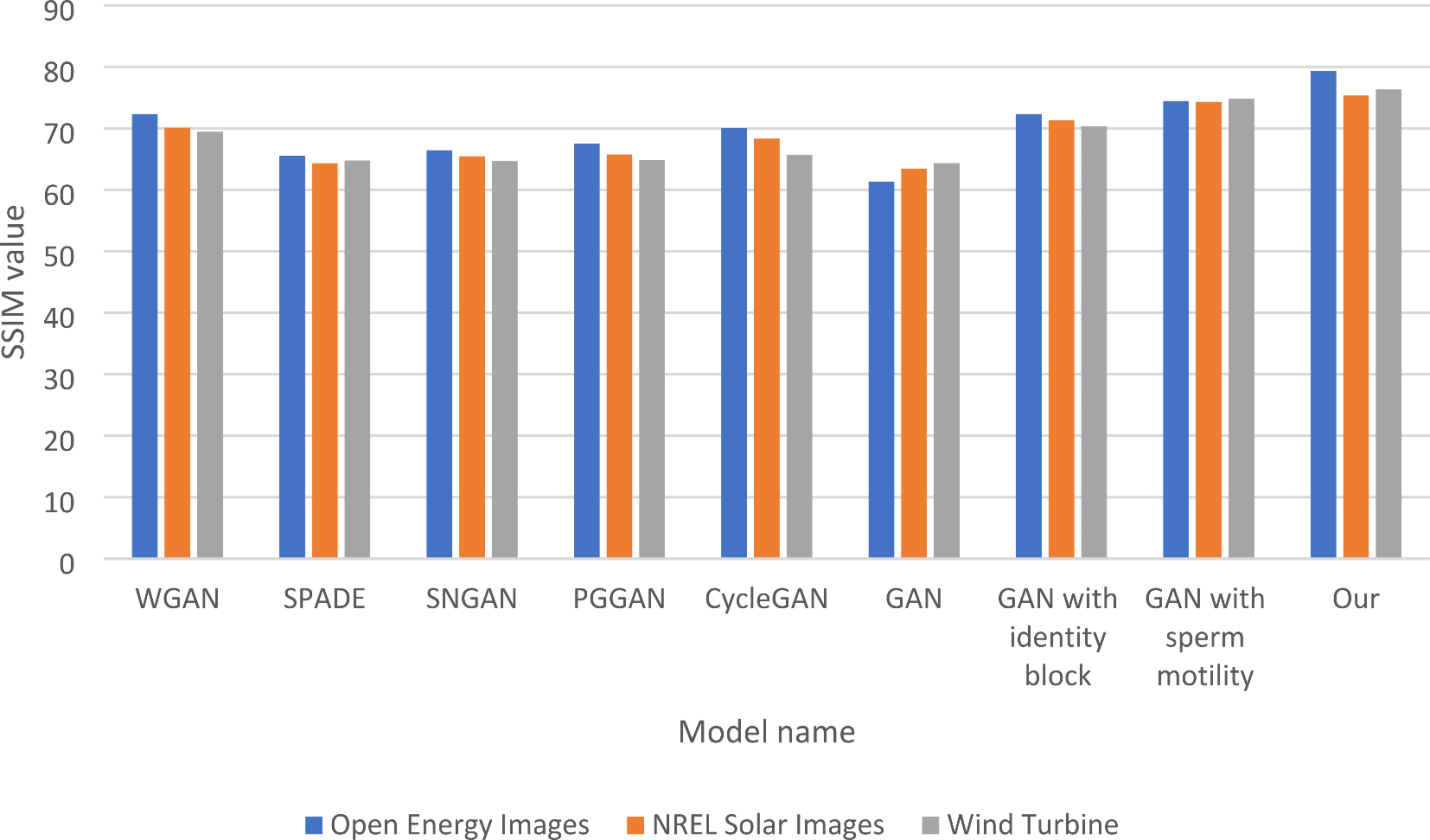
SSIM Comparison of five Models and GSIP-GAN Across three Datasets.

Meanwhile, the GAN with sperm motility significantly enhances performance, achieving SSIM scores of 74.43 for Open Energy Images and 74.83 for Wind Turbine, indicating that this modification effectively improves structural preservation. The findings in Table 4 emphasize the effectiveness of the enhancements implemented in “Our model,” which leads to the highest SSIM scores and demonstrates a robust ability to maintain structural integrity across different datasets. This suggests that integrating advanced techniques can significantly enhance GAN performance, making it a promising approach for future research and applications in image generation. Figure 5 shows the SSIM Comparison of five Models and GSIP-GAN Across three Datasets. The last four groups of columns show the improvement of traditional GANs after adding each part of the integrated model, such as the identity block, sperm mobility, and the complete modifications integrations.

**Table 4:** SSIM comparison of five models and GSIP-GAN across three datasets.

Model	Open energy images	NREL solar images	Wind turbine
Dataset			
WGAN	72.32	70.11	69.43
SPADE	65.55	64.32	64.77
SNGAN	66.43	65.45	64.65
PGGAN	67.53	65.75	64.83
CycleGAN	70.01	68.32	65.65
GAN	61.32	63.43	64.34
GAN with identity block	72.32	71.32	70.32
GAN with sperm motility	74.43	74.32	74.83
Our	79.34	75.33	76.32

### The diversity results

This part of the results shows the comparison between GSIP-GAN and Wasserstein GAN (WGAN), Spectral Normalized GAN (SNGAN), Progressive Growing of GANs (PGGAN), Cycle-Consistent GANs (CycleGAN), and Spatially Adaptive Normalization (SPADE) based on FID and IS to measure the diversity of generation of the missing pixel.

In Table 5, the FID (Fréchet Inception Distance) comparison evaluates the diversity of generated images across three datasets: Open Energy Images, NREL Solar Images, and Wind Turbine. A lower FID score indicates better quality and diversity in the generated samples. “Our model” demonstrates superior performance with the lowest FID scores of 178.23 for Open Energy Images, 178.32 for NREL Solar Images, and 145.45 for Wind Turbine, signifying its ability to generate diverse and high-quality outputs. In contrast, the traditional GAN model exhibits relatively high FID scores, particularly 253.87 for Open Energy Images and 276.09 for NREL Solar Images, indicating significant challenges in producing diverse and realistic images. Other models, such as WGAN and CycleGAN, also show higher FID scores, with WGAN achieving 232.32 for Open Energy Images and 193.43 for NREL Solar Images. These results suggest that while these models perform better than the traditional GAN, they still lack the diversity and quality exhibited by “Our model.“.

**Fig. 6:**
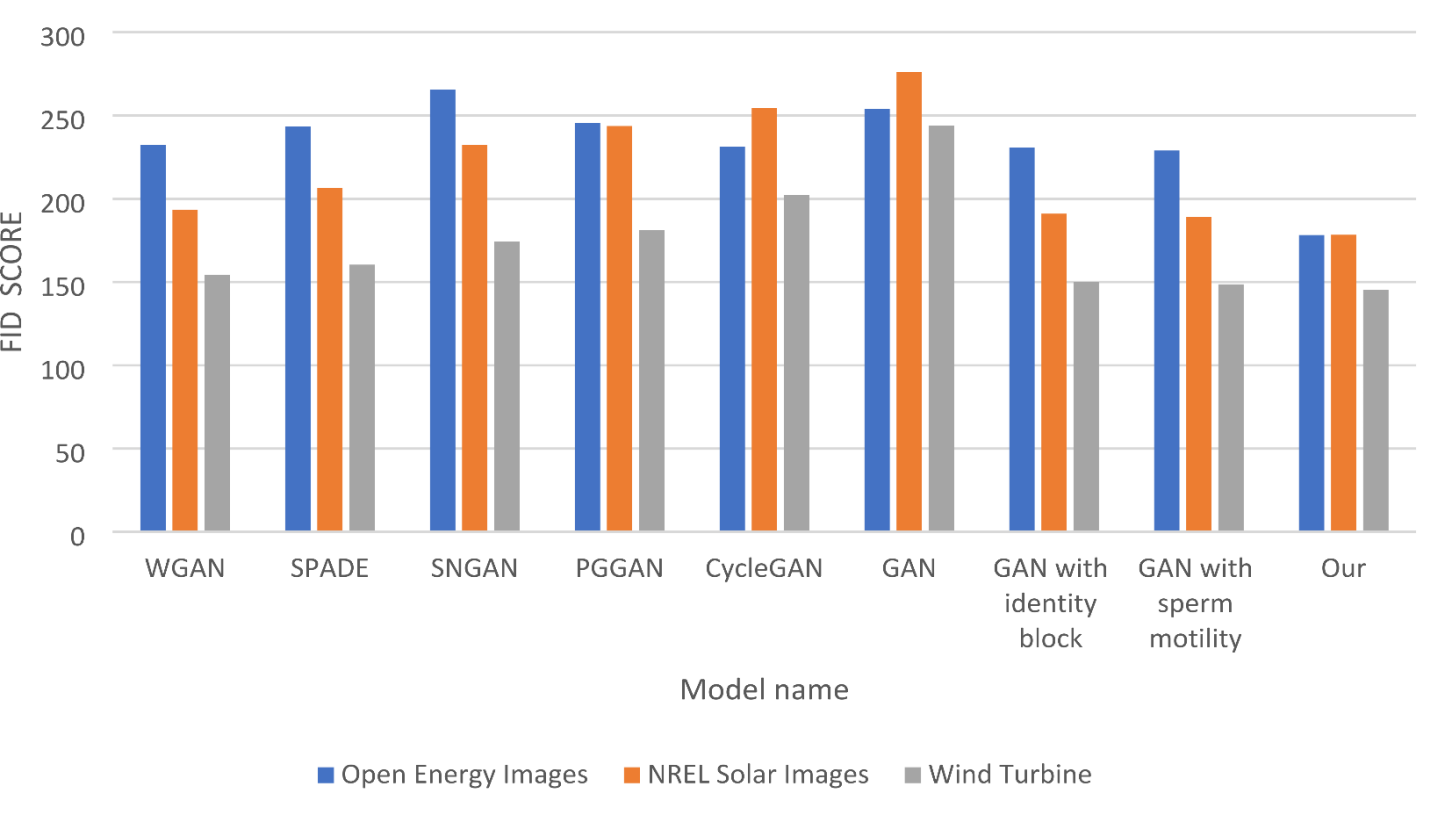
FID Comparison of five Models and GSIP-GAN Across three Datasets.

The ablation tests shed light on the impact of specific enhancements. The GAN with the identity block achieves an FID score of 230.76 for Open Energy Images, which is an improvement over the traditional GAN but still falls short of “Our model.” Similarly, the GAN with sperm motility performs well with scores of 228.96 for Open Energy Images and 189.09 for NREL Solar Images, indicating that these modifications effectively contribute to enhancing diversity in the generated outputs. The findings in Table 5 highlight the effectiveness of the enhancements integrated into “Our model,” which leads to significantly lower FID scores and, consequently, greater diversity in the generated images. This underscores the importance of these advanced techniques in improving the quality and variety of outputs in GAN architectures, making “Our model” a promising approach for future applications in image generation. Figure 6 shows FID Comparison of five Models and GSIP-GAN Across three Datasets and also show the improvement for the traditional GANs after adding the identity block and sperm mobility alone.

**Table 5:** FID comparison of five models and GSIP-GAN across three datasets.

Model	Open energy images	NREL solar images	Wind turbine
Dataset			
WGAN	232.32	193.43	154.34
SPADE	243.43	206.43	160.43
SNGAN	265.43	232.32	174.23
PGGAN	245.45	243.65	181.23
CycleGAN	231.21	254.34	202.21
GAN	253.87	276.09	243.96
GAN with identity block	230.76	190.98	149.98
GAN with sperm motility	228.96	189.09	148.64
Our	178.23	178.32	145.45

**Fig. 7:**
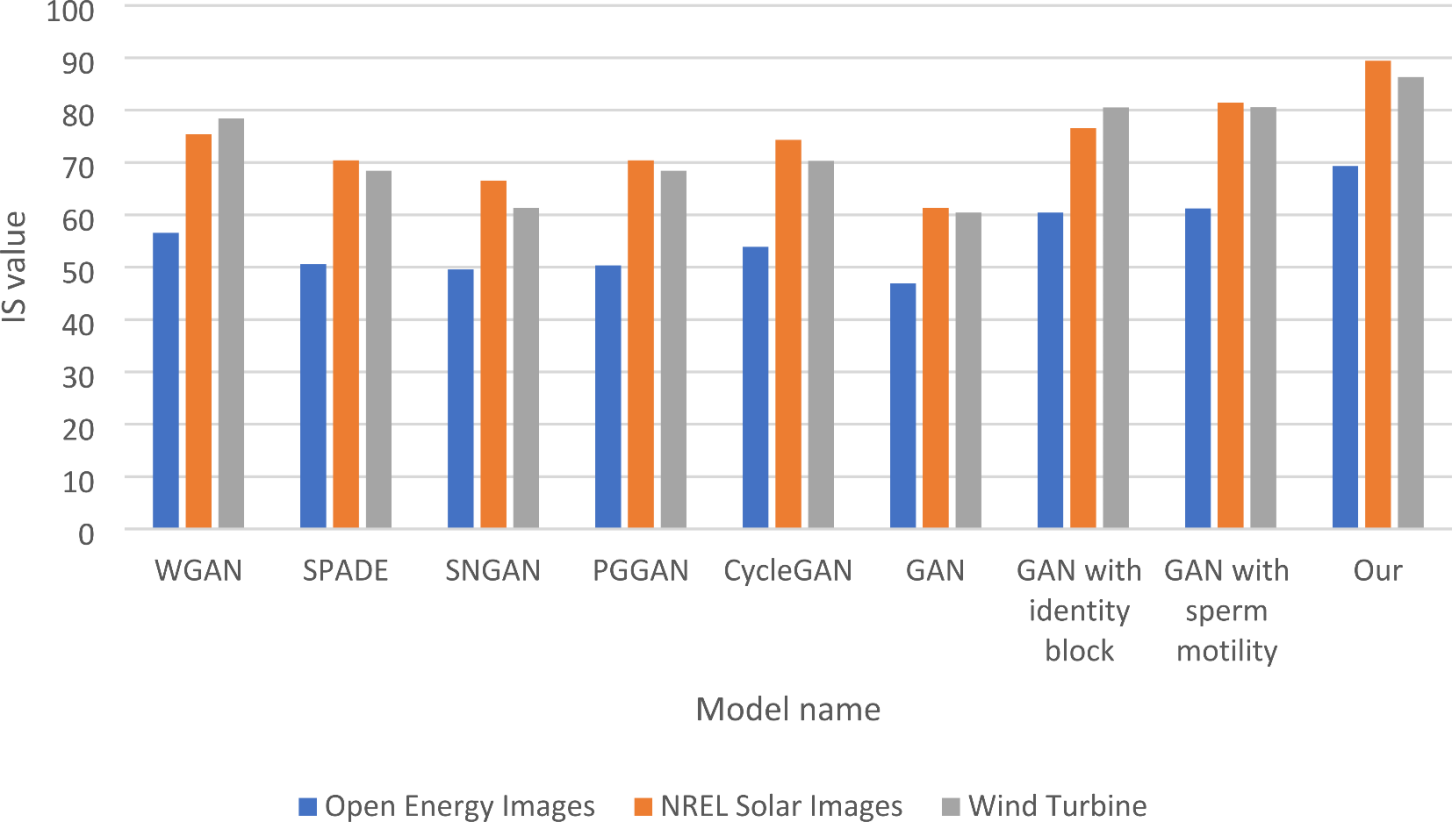
IS Comparison of five Models and GSIP-GAN Across three Datasets.

In Table 6, the IS (Inception Score) comparison evaluates the quality and diversity of generated images across three datasets: Open Energy Images, NREL Solar Images, and Wind Turbine. The results indicate that “Our model” achieves the highest IS scores, with values of 69.32 for Open Energy Images, 89.43 for NREL Solar Images, and 86.34 for Wind Turbine. These scores reflect not only the quality of generated images but also their diversity, demonstrating the model’s ability to produce a wide range of visually distinct outputs. In contrast, the traditional GAN model exhibits the lowest performance, with IS scores of 46.95 for Open Energy Images and 61.32 for NREL Solar Images. This indicates significant limitations in both the quality and diversity of the generated images. Other models, such as WGAN and CycleGAN, show moderate IS scores, with WGAN achieving 56.56 for Open Energy Images and 75.45 for NREL Solar Images, suggesting that while they perform better than the traditional GAN, they still do not match the capabilities of “Our model.” The ablation tests provide further insights into the impact of specific enhancements. The GAN with the identity block scores 60.42 for Open Energy Images and 76.56 for NREL Solar Images, showing a notable improvement over the traditional GAN. Similarly, the GAN with sperm motility achieves scores of 61.23 for Open Energy Images and 81.43 for NREL Solar Images, indicating that these modifications effectively enhance both the quality and diversity of generated images. The findings in Table 6 highlight the effectiveness of the enhancements integrated into “Our model,” which leads to significantly higher IS scores across all datasets. This underscores the importance of advanced techniques in improving the quality and diversity of outputs in GAN architectures, making “Our model” a compelling approach for future applications in image generation. Figure 7 shows the IS Comparison of five Models and GSIP-GAN Across three Datasets and the last four groups of the columns show the enhancement for the traditional GANs after adding the identity block and the sperm mobility.

**Table 6:** IS comparison of five models and GSIP-GAN across three datasets.

Model	Open energy images	NREL solar images	Wind turbine
Dataset			
WGAN	56.56	75.45	78.43
SPADE	50.56	70.43	68.43
SNGAN	49.54	66.54	61.32
PGGAN	50.34	70.43	68.43
CycleGAN	53.88	74.32	70.32
GAN	46.95	61.32	60.43
GAN with identity block	60.42	76.56	80.52
GAN with sperm motility	61.23	81.43	80.54
Our	69.32	89.43	86.34

### Case study of fault detection of solar images

**Fig. 8:**
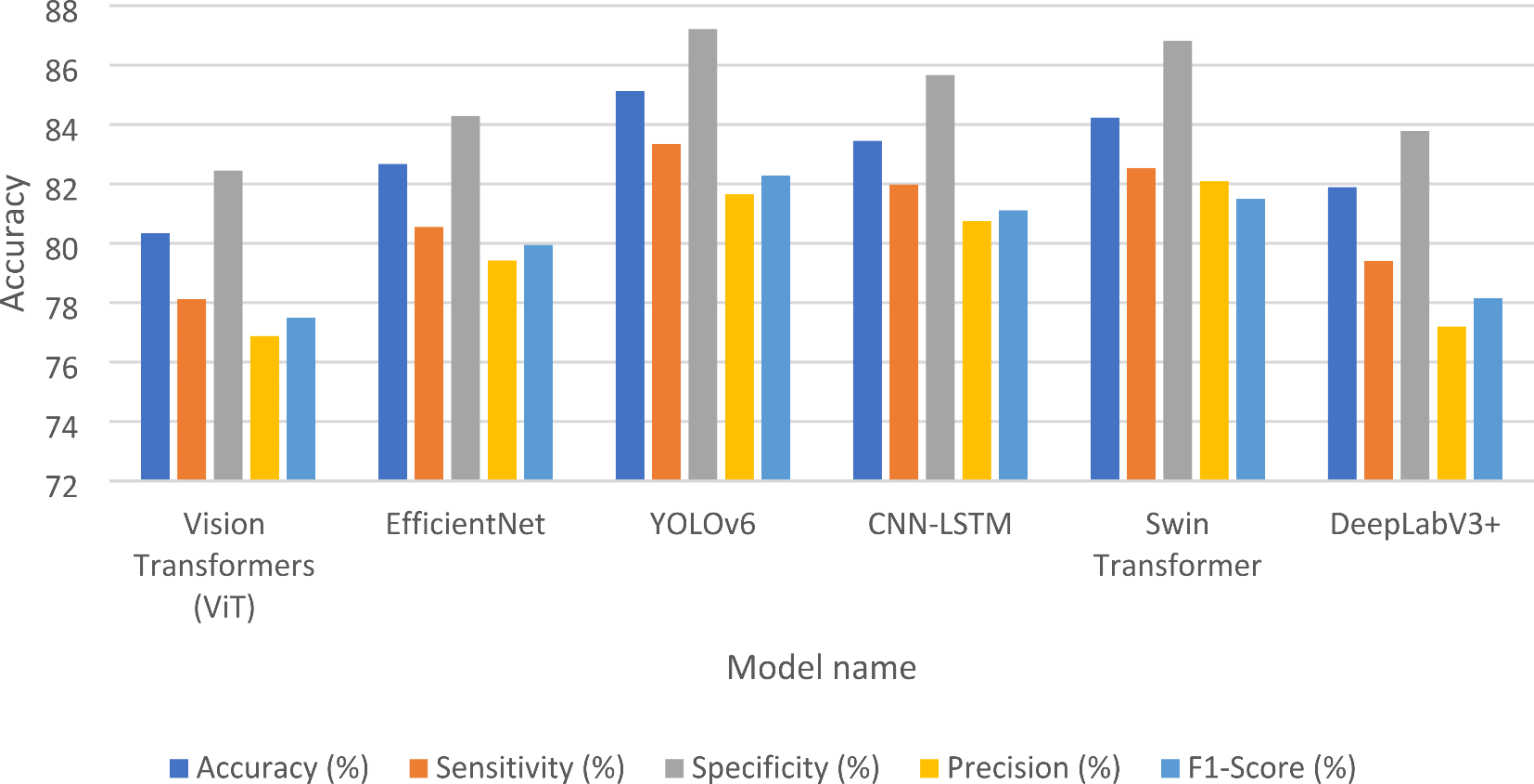
Performance metrics bar chart before using GSIP model for image reconstruction.

**Fig. 9:**
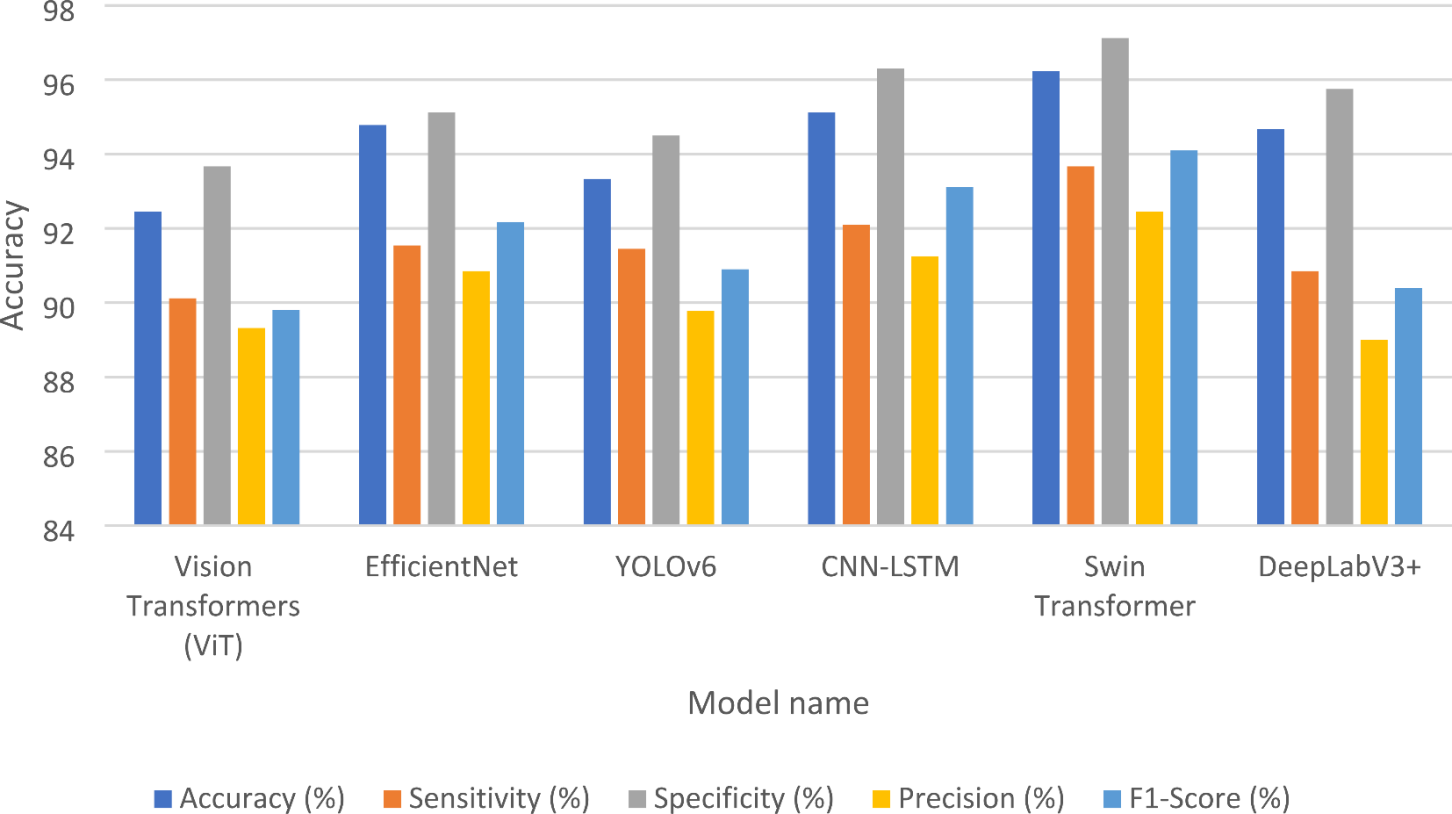
Performance Metrics bar chart After Using GSIP Model for Image Reconstruction.

**Fig. 10:**
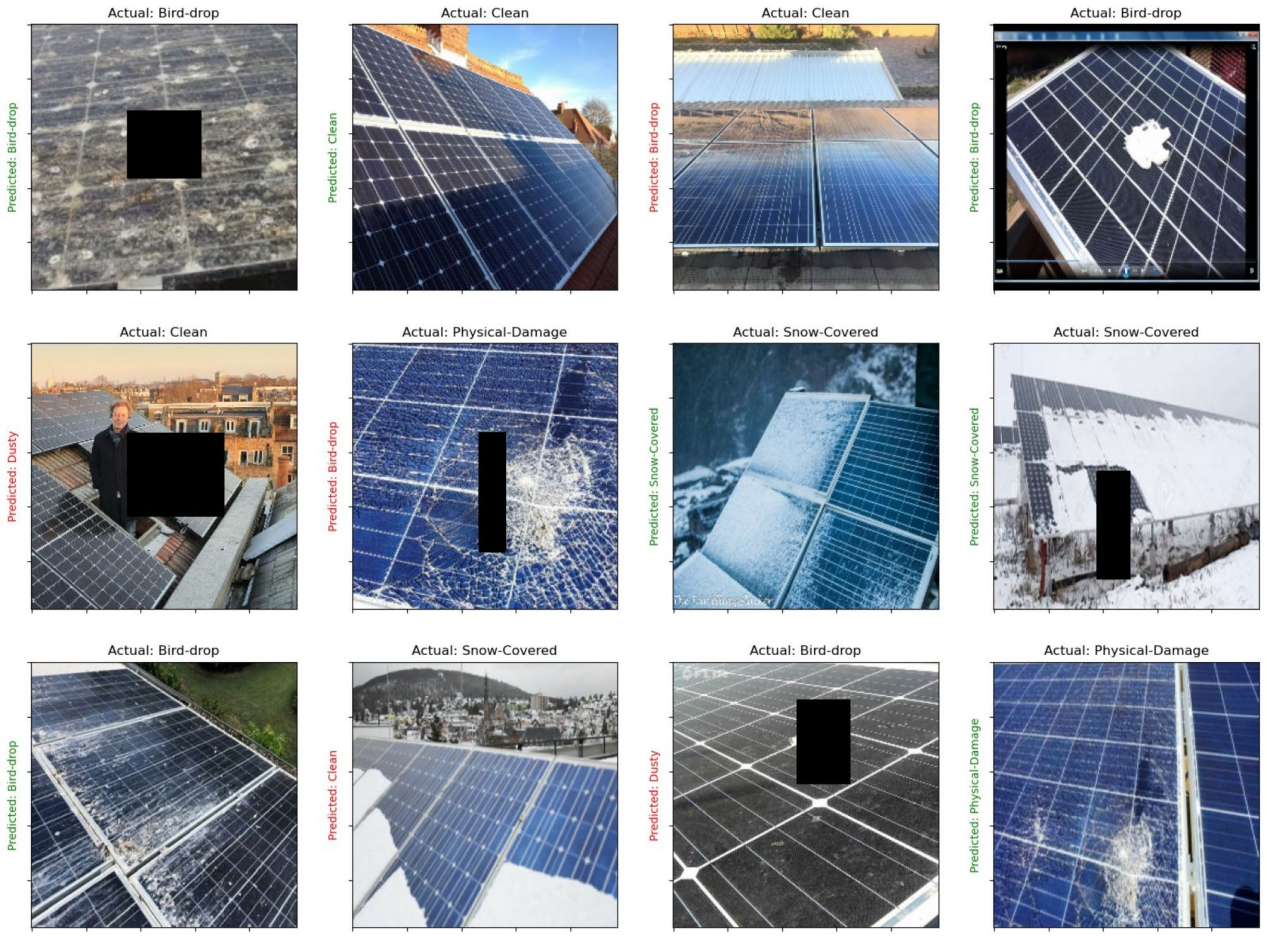
Examples of solar panels fault detection using Swin Transformer before pixel imputation.

**Fig. 11:**
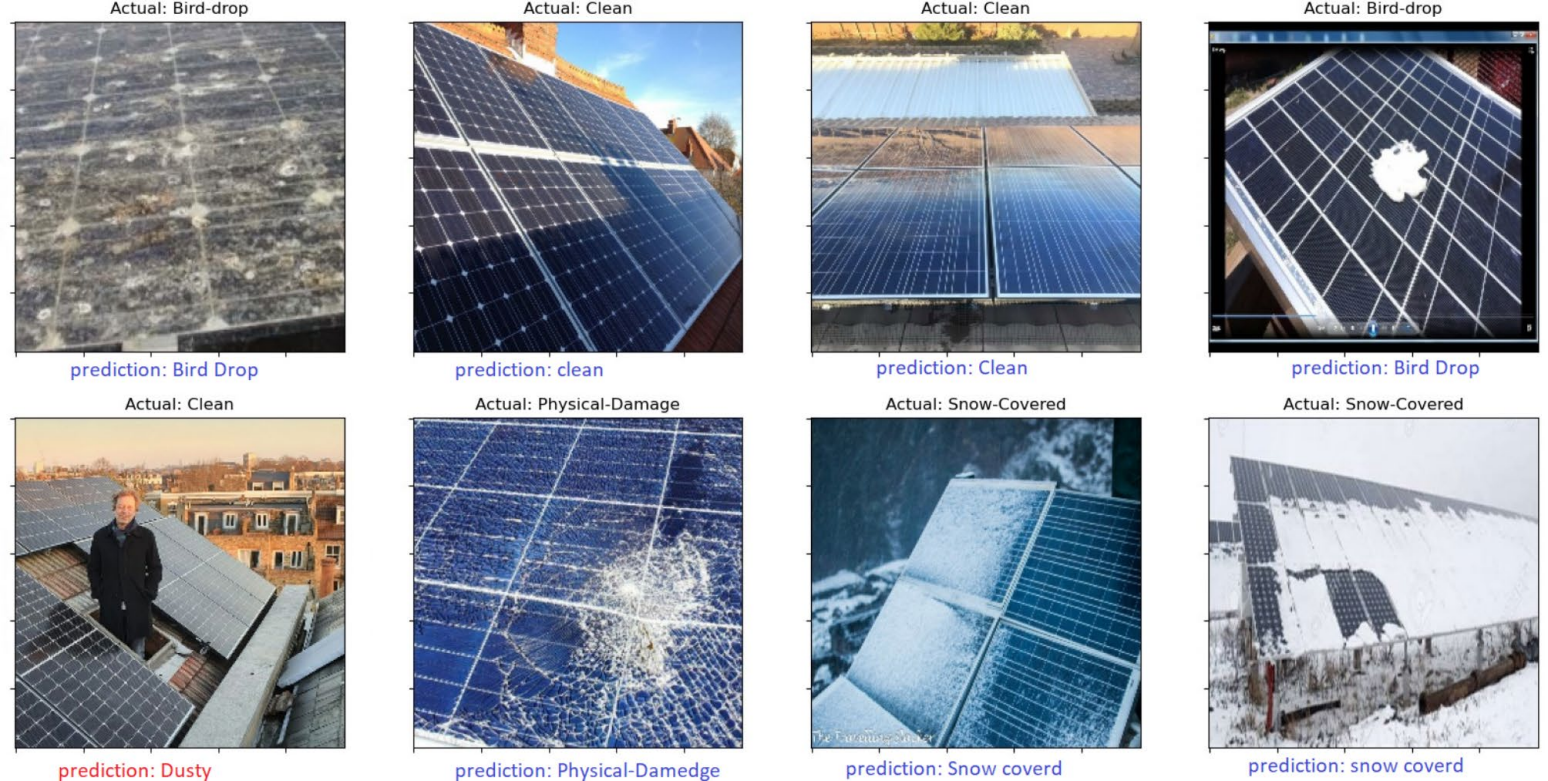
Examples of solar panels fault detection using Swin Transformer after pixel imputation.

This subsection presents case study for detection the fault in images of solar panels before and after using our model in missing pixel imputation and image reconstruction. The performance metrics presented in Table 7 showcase the effectiveness of various models for fault detection in solar panels prior to the application of the GSIP model for image reconstruction. Among the models evaluated, YOLOv6 achieved the highest accuracy at 85.12%, indicating its strong capability in correctly identifying faults. It also exhibited a notable sensitivity of 83.34%, reflecting its ability to correctly identify true positive cases. EfficientNet^36^ and CNN-LSTM^37^ followed closely, with accuracy rates of 82.67% and 83.45%, respectively, demonstrating their robustness in detecting faults while maintaining reasonable sensitivity and specificity levels. Conversely, the Vision Transformers (ViT)^38^ and DeepLabV3+^39^ models showed slightly lower performance metrics, with accuracies of 80.34% and 81.89%. Their sensitivity and precision scores were also lower, suggesting that while these models can be effective, they may require further optimization for practical applications. Overall, the initial performance metrics indicate a solid foundation for fault detection, albeit with room for improvement in sensitivity and precision across most models. After the implementation of the GSIP model for image reconstruction, the results displayed in Table 8 reveal significant enhancements in all performance metrics. The Swin Transformer^40^ emerged as the top performer, achieving an impressive accuracy of 96.23%. This model notably improved its sensitivity to 93.67%, indicating a much higher rate of correctly identifying faults. Similarly, EfficientNet and CNN-LSTM also demonstrated substantial gains, with accuracies of 94.78% and 95.12%, respectively. These improvements suggest that the GSIP model effectively enhances the ability of these architectures to detect faults, likely due to improved feature extraction and noise reduction in the reconstructed images. The overall increase in precision and F1-scores across the board underscores the effectiveness of the GSIP model in bolstering fault detection capabilities. For instance, YOLOv6^41^ improved to 93.33% accuracy with a sensitivity of 91.45%, showcasing its enhanced reliability post-reconstruction. The DeepLabV3 + model also saw an increase in its performance metrics, achieving 94.67% accuracy and a precision of 89.00%. Figure 8 shows the performance Metrics bar chart Before Using GSIP Model for Image Reconstruction in detection the fault in the solar panels and Fig. 9 shows the performance Metrics bar chart after Using GSIP Model for Image Reconstruction in detection the fault in the solar panels. The two figures show the enhancement after using our model for the different models in the detection process. Figure 10 shows the detection process using the swim transformer before using our model in missing pixel imputation and Fig. 11 show the enhancement in the images and the detection results after using our model in missing pixel imputation.

**Table 7:** Performance Metrics before using GSIP Model for Image Reconstruction.

Model Name	Accuracy (%)	Sensitivity (%)	Specificity (%)	Precision (%)	F1-Score (%)
Vision Transformers (ViT)	80.34	78.12	82.45	76.88	77.50
EfficientNet	82.67	80.55	84.29	79.42	79.95
YOLOv6	85.12	83.34	87.21	81.65	82.28
CNN-LSTM	83.45	81.98	85.67	80.75	81.11
Swin Transformer	84.23	82.54	86.82	82.10	81.50
DeepLabV3+	81.89	79.41	83.78	77.20	78.15

**Table 8:** Performance Metrics after using GSIP Model for Image Reconstruction.

Model Name	Accuracy (%)	Sensitivity (%)	Specificity (%)	Precision (%)	F1-Score (%)
Vision Transformers (ViT)	92.45	90.12	93.67	89.32	89.80
EfficientNet	94.78	91.54	95.12	90.85	92.17
YOLOv6	93.33	91.45	94.50	89.78	90.90
CNN-LSTM	95.12	92.10	96.30	91.25	93.11
Swin Transformer	96.23	93.67	97.12	92.45	94.10
DeepLabV3+	94.67	90.85	95.75	89.00	90.40

## The conclusion

This paper introduced a novel GAN-based approach for missing pixel imputation that addresses key challenges in GAN training and pixel selection. The proposed method integrates three key innovations: an identity module to mitigate the vanishing gradient problem, a sperm motility-inspired metaheuristic algorithm to optimize pixel selection, and an adaptive interval mechanism to enhance the generator’s efficiency and coherence of the imputed pixels. Extensive experiments on diverse datasets demonstrated the superior performance of the proposed method in maintaining pixel integrity and addressing common GAN issues like mode collapse. The integration of biological inspiration through the sperm motility heuristic, combined with the architectural enhancements, enabled the system to generate high-quality, contextually accurate pixel imputations even in the presence of high percentages of missing data. The contributions of this work extend beyond just pixel imputation, as the proposed techniques have the potential to benefit a wide range of image processing tasks where robust reconstruction of missing or corrupted data is crucial, such as image enhancement, segmentation, and object detection. The proposed GAN-based approach for missing pixel imputation has demonstrated promising results, but there are several avenues for future research and improvement. Exploring adaptive architectures, extending the framework to video data, incorporating multi-modal data, accelerating inference, and investigating alternative biological inspirations could further enhance the method’s adaptability, generalization, and practical applicability. Adapting the framework to handle missing pixel imputation in video sequences could enable its application to a broader range of multimedia processing tasks, such as video restoration and anomaly detection. Integrating additional data modalities, like depth information or semantic context, could potentially improve the accuracy and robustness of the pixel imputation process. Investigating optimization techniques or hardware-specific implementations to improve the computational efficiency of the inference process could make the proposed method more suitable for real-time or resource-constrained applications. Exploring other biologically inspired mechanisms, beyond sperm motility, and their integration into the GAN framework could lead to new insights and advancements in image processing.

## Data Availability

The datasets used and/or analyzed during the current study are available from the corresponding author upon reasonable request.
